# Pre-Transplant Natural Killer Cell Activity Predicts Survival and Tumor Recurrence After Living Donor Liver Transplantation

**DOI:** 10.3390/jcm15062258

**Published:** 2026-03-16

**Authors:** Eui Soo Han, Ho Joong Choi, Jin Ha Chun, Jiyoung Kim, Young Kyoung You

**Affiliations:** Department of Surgery, Seoul St. Mary’s Hospital, College of Medicine, The Catholic University of Korea, Seoul 06591, Republic of Korea; uishann@gmail.com (E.S.H.); jinhazz@naver.com (J.H.C.); ajykim7@gmail.com (J.K.); youyoungkyoung@gmail.com (Y.K.Y.)

**Keywords:** natural killer cell, hepatocellular carcinoma, living donor liver transplantation, immunologic biomarker, recurrence, survival

## Abstract

**Background/Objectives**: Natural killer (NK) cells are essential mediators of innate immune defense and play a key role in tumor surveillance following liver transplantation (LT). Despite this, the prognostic relevance of pre-transplant NK cell activity in living donor LT (LDLT) has not been fully established. **Methods**: This retrospective analysis included 134 adult patients who underwent LDLT. NK cell activity was evaluated prior to transplantation using interferon-γ release assays and classified as low (<10 pg/mL) or high (≥10 pg/mL). Overall survival (OS) was assessed for all participants, whereas recurrence-free survival (RFS) was analyzed patients with HCC. **Results**: Patients classified as having high NK activity (≥10 pg/mL) experienced significantly better overall survival compared to those with low activity (log-rank *p* = 0.032). In the multivariate analysis, high NK activity showed a strong trend toward improved overall survival (HR, 0.52; 95% CI, 0.26–1.04; *p* = 0.063). Among recipients with HCC, high NK activity was associated with a markedly improved recurrence-free survival (log-rank *p* = 0.004). Multivariate Cox regression further established NK activity as an independent factor for tumor recurrence (HR, 0.27; 95% CI, 0.08–0.87; *p* = 0.028). **Conclusions**: Pre-transplant NK cell activity independently predicts both survival and recurrence following LDLT.

## 1. Introduction

Liver transplantation (LT) is the curative standard for end-stage liver disease and selected hepatocellular carcinoma (HCC) [1,2]. However, long-term survival is frequently compromised by tumor recurrence and immune-related complications, highlighting the need for reliable prognostic markers [3,4]. Among various factors, the recipient’s innate immune competence—particularly the functional status of natural killer (NK) cells—is critical for graft protection and tumor immunosurveillance [5,6,7].

NK cells act as the first line of defense, mediating direct cytotoxicity against malignant cells and orchestrating downstream adaptive immune responses [7,8,9]. While many studies have focused on the recovery of NK cell function after transplantation, the clinical relevance of a recipient’s baseline immune fitness prior to surgery remains poorly defined [10,11,12]. In the setting of Living Donor Liver Transplantation (LDLT), the predictable timing of the procedure makes such preoperative assessment particularly actionable, allowing for risk stratification before the initiation of intensive postoperative immunosuppression. This preoperative status is especially vital in LT recipients, as the initiation of intensive postoperative immunosuppression can abruptly alter the immune landscape, potentially masking or exacerbating underlying deficiencies [13,14].

Consequently, identifying patients with low preoperative NK activity could allow for more personalized risk stratification and adjusted management strategies. In this study, we investigated the prognostic significance of pre-transplant NK cell activity in living donor liver transplantation (LDLT). We hypothesized that higher baseline NK activity indicates superior immune competence, which independently predicts improved overall survival (OS) and reduced recurrence-free survival (RFS) in HCC patients.

## 2. Materials and Methods

This retrospective cohort study enrolled 134 consecutive adult patients who received living donor liver transplantation (LDLT) at Seoul St. Mary’s Hospital, The Catholic University of Korea, between January 2020 and December 2023. The recruitment period and the consecutive nature of patient enrollment have been explicitly stated to enhance transparency. All demographic and clinical variables were extracted from the institutional database and further validated through a comprehensive manual chart review to ensure data accuracy and minimize information bias. The cohort size represented all qualifying cases performed during this timeframe; a post hoc power calculation indicated approximately 82% power to detect a hazard ratio of 0.5 for overall survival with a two-sided α of 0.05. All transplant procedures were conducted following uniform surgical protocols by a single hepatobiliary surgical team. Exclusion criteria were age below 18 years and incomplete pre-transplant immune data, perioperative records, or follow-up documentation. Demographic and clinical variables for both recipients and donors, as well as intraoperative factors and post-transplant outcomes, were extracted from the institutional LT database and validated through manual chart review.

Pre-transplant peripheral blood samples were collected within two weeks prior to surgery to evaluate NK cell activity. NK cell function was measured using the NK Vue^®^ interferon-γ release assay (ATGen, Seongnam, Republic of Korea), which quantifies IFN-γ secretion after stimulation of peripheral NK cells with Promoca^®^ (a mixture of recombinant cytokines, including IL-12, IL-15, and IL-18, specifically designed to stimulate NK cells). Results were reported as IFN-γ concentration (pg/mL), and patients were categorized into low (<10 pg/mL) and high (≥10 pg/mL) NK activity groups, in alignment with both the manufacturer’s threshold and previous studies assessing immunologic competence in solid organ transplantation. This cutoff value has also been validated in earlier oncologic and transplant research [13].

The initial immunosuppression protocol did not differ between HCC patients and other benign diseases. Immunosuppressants included triple regimen of a calcineurin inhibitor (Cyclosporine or Tacrolimus), mycophenolate mofetil, and steroids. An interleukin-2 receptor blocker was administered on both the day of the operation and the fourth postoperative day. Steroids were withdrawn 1 month after surgery, and mycophenolate mofetil was withdrawn 6 months after surgery. Only a low dose of a single calcineurin inhibitor was administered after this period.

Overall survival (OS) was defined as the time from transplantation to death from any cause or last follow-up, whereas recurrence-free survival (RFS) was assessed exclusively among patients who underwent transplantation for hepatocellular carcinoma (HCC), defined as the interval from transplantation to radiologic evidence of recurrence or death, whichever occurred first. Patients alive without recurrence were censored at the date of their last surveillance imaging. Diagnosis of HCC recurrence was based on radiologic criteria using multiphasic CT or MRI following LI-RADS guidelines, with confirmation by multidisciplinary consensus when necessary.

Comparative analyses were conducted between low- and high-NK groups. Continuous variables were presented as mean ± standard deviation (SD) or median (interquartile range) where appropriate, and categorical variables as absolute counts and percentages. Differences between groups were evaluated using Student’s *t*-test or Mann–Whitney U-test for continuous data, and chi-square or Fisher’s exact test for categorical data. Survival analyses employed the Kaplan–Meier method, with differences between survival curves assessed using the log-rank test. Univariate Cox proportional hazards models were used to identify potential predictors of OS and RFS; variables with *p* < 0.30 were included in multivariate analyses via the enter method. Hazard ratios (HRs) and associated 95% confidence intervals (CIs) were calculated to estimate the relative risk for each variable. The proportional hazards assumption for the Cox models was verified using Schoenfeld residual tests. Multicollinearity among covariates was assessed using Variance Inflation Factors (VIF).

All statistical analyses were conducted using IBM SPSS Statistics version 26.0 (IBM Corp., Armonk, NY, USA). A two-tailed *p*-value < 0.05 was considered to indicate statistical significance.

## 3. Results

A total of 134 adult patients who underwent living donor liver transplantation (LDLT) were included in this analysis. The median follow-up period was 41 months (range, 6–67 months). Based on pre-transplant NK cell activity, 56 patients (41.8%) were categorized as the low NK group (<10 pg/mL), while 78 patients (58.2%) composed the high NK group (≥10 pg/mL). Baseline clinical characteristics are detailed in Table 1. There were no statistically significant differences between groups regarding age, sex, body mass index (BMI), diabetes, or hypertension. Additionally, no difference was found in the etiology of liver disease, donor age, graft type, graft-to-recipient weight ratio (GRWR), or intraoperative parameters. Nevertheless, the high-NK group exhibited lower MELD scores (12.3 ± 7.1 vs. 18.9 ± 9.5, *p* < 0.001) and lower serum CRP levels (0.5 ± 1.1 vs. 1.8 ± 2.6, *p* < 0.001). The incidence of postoperative cytomegalovirus (CMV) infection was also significantly lower in the high-NK group (14.1% vs. 43.6%, *p* < 0.001).

Among the 70 patients who underwent transplantation due to hepatocellular carcinoma (HCC), subgroup characteristics are presented in Table 2. There were no significant differences between the NK groups in terms of age, sex, BMI, pre-transplant tumor markers (AFP, PIVKA-II), or tumor size. MELD and CRP values remained significantly lower in the high-NK group (*p* = 0.001 for both). Recurrence of HCC was observed in 39.1% of low-NK patients and 10.6% of high-NK patients (*p* = 0.013), and the frequency of CMV infection was notably higher in the low-NK group (43.5% vs. 6.4%, *p* < 0.001).

**Figure 1:**
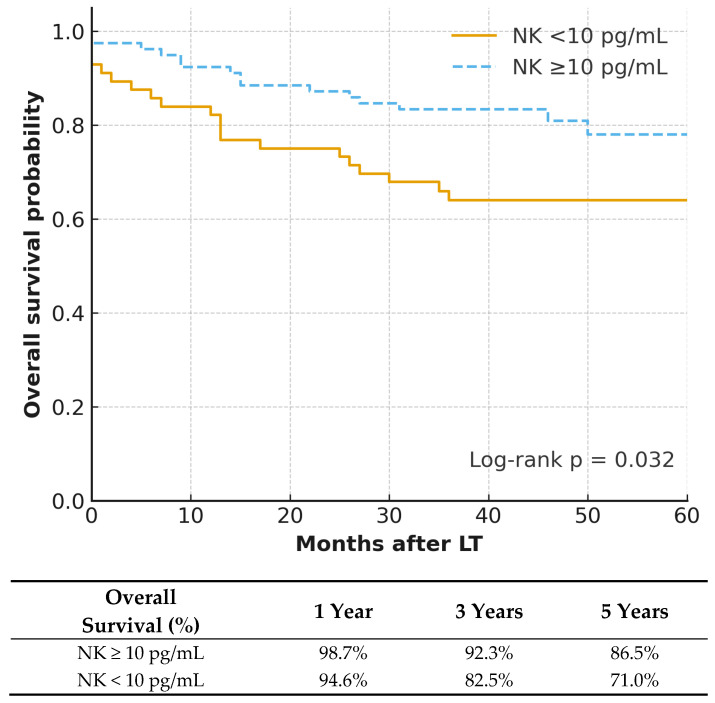
Overall Survival Stratified by Pre-transplant NK Cell Activity. Kaplan–Meier analysis demonstrates a significant improvement in overall survival among patients with high NK activity (≥10 pg/mL) compared to those with low NK activity (<10 pg/mL) (log-rank *p* = 0.032). The number at risk is provided below the *X*-axis.

The Kaplan–Meier survival analysis revealed a significant difference in survival outcomes between the NK groups (Figure 1). The 1-, 3-, and 5-year overall survival rates were 94.6%, 82.5%, and 71.0% in the low-NK group compared with 98.7%, 92.3%, and 86.5% in the high-NK group (log-rank *p* = 0.032). The proportional hazards assumption was not violated for any variables in the OS model (global *p* = 0.412). In multivariate Cox regression analysis (Table 3), high NK activity (≥10 pg/mL) showed a trend toward improved overall survival, although it did not reach absolute statistical significance (HR 0.52, 95% CI 0.26–1.04, *p* = 0.063). None of the other covariates in the multivariate model, including MELD ≥ 25 (*p* = 0.959), presence of HCC (*p* = 0.586), or non-HBV etiology (*p* = 0.531), had a significant effect on survival.

**Figure 2:**
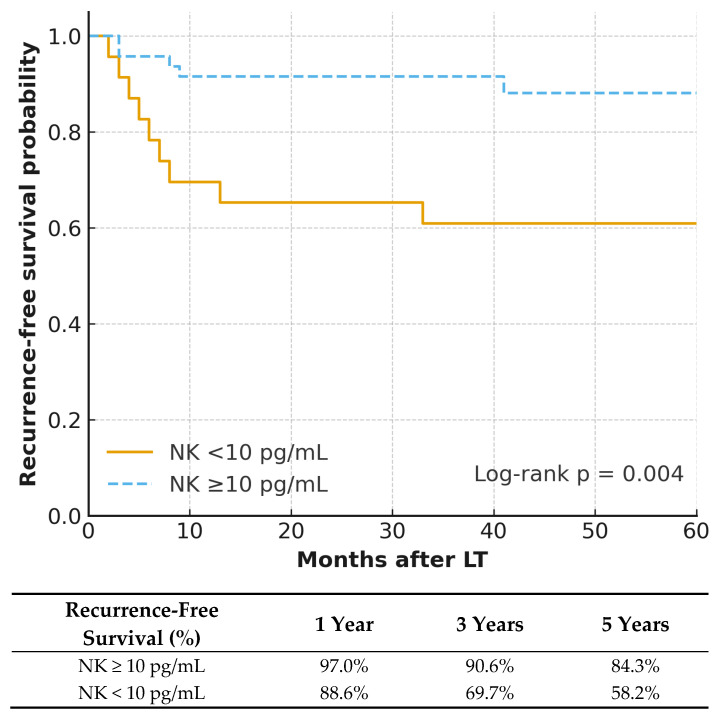
Recurrence-free Survival in HCC Recipients Based on NK Cell Activity. High NK activity (≥10 pg/mL) correlated with better recurrence-free survival following liver transplantation (log-rank *p* = 0.004). The number at risk is provided below the *X*-axis.

Within the HCC subgroup, there was a substantial difference in recurrence-free survival (RFS) between NK groups (Figure 2). The 1-, 3-, and 5-year RFS rates were 88.6%, 69.7%, and 58.2%, respectively, in the low-NK group vs. 97.0%, 90.6%, and 84.3% in the high-NK group (log-rank *p* = 0.004). Similarly, the proportional hazards assumption for the RFS model was also satisfied (global *p* = 0.528). Multivariate Cox analysis (Table 4) identified high NK activity as an independent protective factor against recurrence (HR 0.27, 95% CI 0.08–0.87, *p* = 0.028); tumor size ≥ 5 cm significantly increased the risk of recurrence (HR 3.88, 95% CI 1.18–12.69, *p* = 0.025). Other analyzed variables, such as AFP ≥ 200 ng/mL and PIVKA-II ≥ 40 mAU/mL, were not significant in the multivariate model.

## 4. Discussion

In this single-center cohort of 134 living donor liver transplantation (LDLT) recipients, elevated pre-transplant NK cell activity (≥10 pg/mL by IFN-γ release) correlated with improved clinical outcomes. NK cells are crucial effectors within innate immunity, responsible for both viral elimination and tumor immune surveillance, and mounting evidence suggests that their functional status profoundly impacts outcomes following organ transplantation [5,7,15,16]. In the present cohort, individuals exhibiting high NK activity achieved significantly greater overall survival (OS) as demonstrated by Kaplan–Meier analysis (log-rank *p* = 0.032), While the multivariable Cox regression for OS yielded a *p*-value of 0.063 (HR 0.52, 95% CI 0.26–1.04), this substantial 48% reduction in mortality risk represents a strong clinical trend that supports the role of NK cells in mediating anti-tumor cytotoxicity and suppressing residual malignant cell populations. Crucially, these findings are consistent with prior studies that highlight the role of preoperative immune competence in graft protection and decreased mortality, suggesting that a robust innate immune system is a prerequisite for favorable long-term outcomes after transplantation [11,14,17].

Among the 70 patients transplanted for hepatocellular carcinoma (HCC), those with elevated NK cell activity demonstrated substantially reduced recurrence rates in both unadjusted (log-rank *p* = 0.004) and adjusted analyses (HR 0.27, 95% CI 0.08–0.87, *p* = 0.028), supporting the role of NK cells in mediating anti-tumor cytotoxicity and suppressing residual malignant cell populations [9,18,19]. The prognostic significance of preoperative NK activity likely stems from the effective clearance of circulating tumor cells (CTCs). High baseline NK activity provides a critical ‘first line of defense’ neutralizing CTCs and preventing the formation of micrometastatic lesions [20]. This early-stage immune clearance reduces the risk of long-term recurrence and mortality observed for years after the surgery [21]. Importantly, differences in prognosis between the groups became apparent early after transplantation and continued up to 5 years post-transplant. Furthermore, tumor size ≥ 5 cm independently predicted a higher risk of recurrence (HR 3.88, 95% CI 1.18–12.69, *p* = 0.025), underscoring the established importance of tumor burden in shaping oncologic outcomes after LT [3,22].

These results are consistent with existing biological principles. NK cells play a pivotal role in innate immune surveillance through cytotoxic mechanisms targeting malignant hepatocytes, such as perforin–granzyme exocytosis and Fas/FasL-mediated apoptosis, and they also secrete interferon-γ to potentiate downstream antitumor T cell responses [6,7,23]. Increased preoperative NK functional capacity may indicate a greater host immunological “reserve,” facilitating more complete elimination of residual tumor cells and improved early post-transplant immune control during periods of immunosuppression. Unlike earlier investigations that primarily evaluated NK cell restoration post-transplantation, our findings underscore the prognostic impact of baseline NK activity—prior to the initiation of immunosuppression—on long-term cancer and survival outcomes [5,9,24].

From a clinical perspective, a dichotomous, assay-based NK measure presents notable advantages. First, it is objective, expedient, and feasible to implement using peripheral blood samples, thereby supporting its routine application in preoperative assessment [25,26]. Second, its independent predictive value in multivariable analysis suggests that it provides incremental prognostic insight supplemental to standard clinical and oncologic variables [24,26]. Third, among HCC candidates, combining NK activity with established oncologic variables such as tumor size may enhance preoperative risk stratification: patients exhibiting both low NK activity and larger tumors could require more intensive surveillance protocols, increased imaging frequency, or individualized immunosuppression approaches following transplantation [3,22]. While our study was not intended to evaluate specific management strategies, these findings provide a rationale for developing NK-based pathways in peri-transplant decision-making.

Nonetheless, several limitations must be acknowledged. This study is subject to limitations inherent in its single-center, retrospective design, which may limit the generalizability of our findings to other clinical settings. While we attempted to reduce bias by using a consecutive patient cohort and standardized protocols, the relatively small sample size—particularly in the HCC subgroup (n = 70)—and the lack of external validation may affect the robustness of our results. Future prospective, multicenter studies are necessary to confirm the external validity of preoperative NK activity as a prognostic marker. Furthermore, this study did not include a healthy control group. While establishing a normal reference range is important, our focus was on internal risk stratification for prognostic purposes within the transplant candidate population, rather than defining a physiological baseline for the healthy population. Since all participants shared similar disease backgrounds, these relative differences remain clinically valid for preoperative screening.

Immunosuppression regimens (e.g., tacrolimus vs. mTOR inhibitors) were excluded from the analysis due to incomplete longitudinal data, but their possible influence on post-transplant immunity remains an important consideration. Postoperative immunosuppressants, such as tacrolimus and corticosteroids, are known to impair NK cell function by reducing interferon-γ production and cytotoxic potential [27,28]. In this context, a high preoperative NK activity likely reflects a robust baseline immune reserve that may afford superior residual tumor surveillance during the early post-transplant period, despite the suppressive effects of medication. Whether this benefit is primarily due to the baseline reserve or a faster recovery of immune function warrants further longitudinal investigation.

The number of events—particularly within the HCC subgroup—was relatively small, which can lead to wider confidence intervals and limit the ability to detect subtle associations. Dichotomizing continuous variables, such as NK activity, may reduce the fidelity of the data and obscure underlying non-linear associations. Nevertheless, we utilized the pre-specified 10 pg/mL cutoff—the validated clinical reference value for the NK Vue^®^ assay—to ensure our findings are practical and easily reproducible for preoperative risk stratification in clinical practice. While conducting sensitivity analyses with continuous NK values or alternative cut-points might yield additional insights, maintaining this standardized threshold aligns with established oncologic research and facilitates direct clinical application by providing a clear objective criterion for surgeons. NK activity was assessed at only a single preoperative time point, and longitudinal changes before and after transplantation were not evaluated, preventing assessment of dynamic immunologic alterations or mediation (e.g., whether early post-LT immunity underlies the observed associations). Furthermore, certain pathological risk features, including microvascular invasion and detailed explant characteristics, were not incorporated into the model, which could enhance recurrence risk prediction when available.

Future research should prospectively validate these results in multicenter settings and investigate whether NK activity, analyzed as a continuous variable and integrated with tumor metrics (size, number, biomarkers), can inform a robust nomogram for individualized risk assessment. These results have the potential to optimize peri-transplant immune monitoring strategies and facilitate the identification of patients eligible for targeted immunomodulatory interventions. Studies that incorporate serial NK measurements alongside immunosuppression data and patterns of infection could provide further insights into mechanisms and help determine optimal intervention periods. The question of whether preservation or enhancement of NK function before or shortly after LT leads to improved cancer outcomes warrants focused investigation. In this context, recent research has highlighted the potential for modulating the HCC immune microenvironment to improve clinical outcomes. For instance, Ginsenoside Rh1 has been demonstrated to regulate the tumor immune landscape via the glucocorticoid receptor, thereby enhancing the efficacy of systemic therapies [29]. Such strategies complement our findings by providing a mechanistic framework for how baseline immune status can be optimized to improve long-term prognosis.

## 5. Conclusions

Despite its limitations, this study has significance to evaluate the possibility that preoperative NK cell activity can be used as a selection criterion for LT recipients. In conclusion, pre-transplant NK cell activity serves as an independent predictor of both overall survival and recurrence in LDLT recipients. When considered alongside tumor size in HCC patients, NK activity provides distinct risk information—reflecting both host immune function and tumor burden—and may be a practical, preoperative biomarker to refine risk stratification and direct post-transplant management.

## Figures and Tables

**Table 1:** Baseline and perioperative characteristics stratified by NK cell activity.

Variable	NK Cell < 10 pg/mL (*n* = 56)	NK Cell ≥ 10 pg/mL (*n* = 78)	*p*-Value
**Recipient Factors**			
Age (years)	54.0 ± 11.2	56.5 ± 7.9	0.136
Sex, Male (%)	37 (66.1%)	57 (73.1%)	0.495
Etiology of disease			0.308
HBV (%)	31 (55.4%)	46 (59.0%)	
HCV (%)	1 (1.8%)	3 (3.8%)	
Alcohol use (%)	14 (25.0%)	23 (29.5%)	
Other causes (%)	10 (17.9%)	6 (7.7%)	
BMI (kg/m^2^)	24.3 ± 4.0	24.3 ± 4.2	0.922
Diabetes mellitus (%)	14 (25.0%)	23 (29.5%)	0.706
Hypertension (%)	16 (28.6%)	24 (30.8%)	0.934
MELD score	18.9 ± 9.5	12.3 ± 7.1	<0.001
Child–Pugh score	8.6 ± 2.9	7.1 ± 2.4	<0.001
CRP (mg/dL)	1.8 ± 2.6	0.5 ± 1.1	<0.001
eGFR (mL/min/1.73 m^2^)	95.4 ± 40.4	92.9 ± 23.5	0.655
HCC present (%)	23 (41.1%)	47 (60.3%)	0.044
**Donor or** **operative characteristics**			
GRWR	1.2 ± 0.3	1.3 ± 1.2	0.474
Duration of operation (min)	426.9 ± 88.4	442.0 ± 107.8	0.391
PRC transfusion (units)	9.6 ± 7.8	7.2 ± 8.0	0.082
FFP transfusion (units)	7.9 ± 6.3	6.2 ± 6.3	0.121
Right liver graft (%)	41 (73.2%)	57 (73.1%)	1.000
**Post-LT clinical outcomes**			
CMV infection rate (%)	24 (43.6%)	11 (14.1%)	<0.001

Values are reported as mean ± SD or n (%). The Student’s *t*-test, chi-square test, or Fisher’s exact test was applied as appropriate.

**Table 2:** HCC subgroup analysis: recipient-related factors, tumor characteristics, and clinical outcomes by NK cell activity.

Variable	NK Cell < 10 pg/mL (HCC+, n = 23)	NK Cell ≥ 10 pg/mL (HCC+, n = 47)	*p*-Value
**Recipient-related factors**			
Age (years)	60.7 ± 7.2	58.3 ± 6.5	0.170
Sex, Male (%)	22 (95.7%)	40 (85.1%)	0.257
BMI (kg/m^2^)	24.1 ± 3.6	24.8 ± 4.5	0.524
Diabetes mellitus (%)	6 (26.1%)	13 (27.7%)	1.000
Hypertension (%)	9 (39.1%)	17 (36.2%)	1.000
MELD score	14.7 ± 8.6	9.7 ± 3.7	0.001
Child–Pugh score	6.9 ± 2.5	6.0 ± 1.8	0.073
CRP (mg/dL)	2.0 ± 3.2	0.3 ± 0.6	0.001
eGFR (mL/min/1.73 m^2^)	87.4 ± 22.3	95.1 ± 13.9	0.080
**Oncologic characteristics**			
AFP (ng/mL)	80.9 ± 182.3	68.0 ± 282.6	0.847
PIVKA-II (mAU/mL)	342.1 ± 509.1	156.6 ± 833.6	0.373
Tumor diameter (cm)	3.7 ± 1.9	2.9 ± 2.0	0.181
**Outcomes following LT**			
CMV infection (%)	10 (43.5%)	3 (6.4%)	<0.001
HCC recurrence (%)	9 (39.1%)	5 (10.6%)	0.013

Values are reported as mean ± SD or n (%). The Student’s *t*-test, chi-square test, or Fisher’s exact test was applied as appropriate.

**Table 3:** Univariate and Multivariate Analyses of Overall Survival.

Parameter	Univariate HR (95% CI)	Univariate *p*-Value	Multivariate HR (95% CI)	Multivariate *p*-Value
NK ≥ 10 pg/mL (vs. <10)	0.49 (0.25–0.96)	0.036	0.52 (0.33–1.04)	0.063
Age ≥ 60 (vs. <60)	1.40 (0.72–2.73)	0.317	–	–
Male (vs. Female)	0.88 (0.43–1.79)	0.715	–	–
MELD ≥ 25 (vs. <25)	1.54 (0.70–3.40)	0.280	1.02 (0.43–2.45)	0.959
HCC present (vs. absent)	0.63 (0.32–1.22)	0.170	0.80 (0.35–1.80)	0.586
BMI ≤ 25 (vs. >25)	1.19 (0.60–2.37)	0.616	–	–
DM or HTN (vs. none)	0.81 (0.41–1.59)	0.543	–	–
Non-HBV etiology (vs. HBV)	1.49 (0.77–2.88)	0.242	1.28 (0.59–2.77)	0.531
eGFR < 60 (vs. ≥60)	1.36 (0.48–3.87)	0.559	–	–

Values are reported as mean ± SD or n (%). The Student’s *t*-test, chi-square test, or Fisher’s exact test was applied as appropriate. Cox proportional hazards models were implemented. The multivariate model includes variables that demonstrated *p* < 0.30 in univariate analysis.

**Table 4:** Recurrence-free Survival Following Liver Transplantation in Patients with Hepatocellular Carcinoma.

Variable	Univariate HR (95% CI)	Univariate *p*-Value	Multivariate HR (95% CI)	Multivariate *p*-Value
NK ≥ 10 pg/mL (vs. <10)	0.23 (0.08–0.69)	0.009	0.27 (0.08–0.87)	0.028
Tumor size ≥ 5 cm (vs. <5 cm)	4.05 (1.26–12.99)	0.019	3.88 (1.18–12.69)	0.025
AFP ≥ 200 ng/mL (vs. <200)	2.18 (0.49–9.77)	0.310	–	–
PIVKA-II ≥ 40 mAU/mL (vs. <40)	2.56 (0.90–7.30)	0.079	1.47 (0.47–4.61)	0.508
Age ≥ 60 (vs. <60)	1.01 (0.35–2.88)	0.985	–	–
MELD ≥ 25 (vs. <25)	N/A	N/A	–	–

Univariate and multivariate Cox proportional hazards analyses were conducted. Variables with a *p* < 0.30 on univariate analysis and those deemed clinically relevant for NK activity were incorporated into the multivariate model. MELD ≥ 25 was excluded from the Cox regression analysis because no recurrence events occurred within this subgroup (n = 2).

## Data Availability

No new data were created, and some data are unavailable due to privacy or ethical restrictions.

## Abbreviations

The following abbreviations are used in this manuscript:

AFP	Alpha-fetoprotein
CI	Confidence interval
DM	Diabetes mellitus
HBV	Hepatitis B virus
HCC	Hepatocellular carcinoma
HTN	Hypertension
HR	Hazard ratio
MELD	Model for End-stage Liver Disease
NK	Natural killer
PIVKA-II	Protein induced by vitamin K absence or antagonist-II
